# A critical role for endocytosis in Wnt signaling

**DOI:** 10.1186/1471-2121-7-28

**Published:** 2006-07-06

**Authors:** Jeremy T Blitzer, Roel Nusse

**Affiliations:** 1 Howard Hughes Medical Institute and Department of Developmental Biology, Stanford University School of Medicine, Stanford, California 94305, USA

## Abstract

**Background:**

The Wnt signaling pathway regulates many processes during embryonic development, including axis specification, organogenesis, angiogenesis, and stem cell proliferation. Wnt signaling has also been implicated in a number of cancers, bone density maintenance, and neurological conditions during adulthood. While numerous Wnts, their cognate receptors of the Frizzled and Arrow/LRP5/6 families and downstream pathway components have been identified, little is known about the initial events occurring directly after receptor activation.

**Results:**

We show here that Wnt proteins are rapidly endocytosed by a clathrin- and dynamin-mediated process. While endocytosis has traditionally been considered a principal mechanism for receptor down-regulation and termination of signaling pathways, we demonstrate that interfering with clathrin-mediated endocytosis actually blocks Wnt signaling at the level of β-catenin accumulation and target gene expression.

**Conclusion:**

A necessary component of Wnt signaling occurs in a subcellular compartment distinct from the plasma membrane. Moreover, as internalized Wnts transit partially through the transferrin recycling pathway, it is possible that a "signaling endosome" serves as a nexus for activated Wnt pathway components.

## Background

The Wnt signaling pathway is largely conserved from *Drosophila *to humans and has been shown to regulate diverse embryonic processes including axis specification and organogenesis. Wnt signaling events have also been implicated in a number of cancers, bone density phenotypes, and neurological conditions in adulthood [1,2]. Wnts are a family of secreted proteins which are glycosylated and lipidated, exerting their predominant physiological effects through receptors of the Frizzled and Arrow/low-density lipoprotein (LDL) receptor-related protein 5/6 (LRP5/6) families. Frizzleds are seven transmembrane domain-containing proteins that are the primary receptors for Wnts, whereas LRPs (specifically LRP5 and LRP6) are single pass membrane proteins that serve as co-receptors. In the "canonical Wnt pathway," Wnts bind to their cognate receptors and stimulate the cytoplasmic stabilization of β-catenin through the inhibition of an elaborate degradation complex consisting of APC, Axin, and glycogen synthase kinase 3 (GSK3). Stabilized β-catenin translocates to the nucleus where it interacts with transcriptional regulators of the T cell factor/lymphoid enhancer factor (TCF/LEF) family of proteins to mediate transcription of Wnt target genes [2]. Recently, there has been evidence suggesting that heterotrimeric G proteins may have a role in Wnt signaling [3,4].

While Wnts, their receptors, and numerous downstream components have been identified through genetic and biochemical approaches, the upstream molecular events occurring directly after Frizzled activation have not been well-characterized. Importantly, it has not been established whether the majority of Wnt signal transduction occurs at the plasma membrane or on some intracellular organelle. There have been very few studies examining Wnt trafficking to intracellular compartments, and those demonstrating internalization of Wg/Frizzled2 in *Drosophila *[5] and Wnt5A/Frizzled4 in mammalian cells [6] have primarily suggested a role for Wnt trafficking in degradation of the ligand/receptor complex following signal transduction. While endocytosis of ligand/receptor pairs has traditionally been considered a longer-term mechanism for termination of a signaling pathway, recent evidence with receptor tyrosine kinases (RTKs) and G protein-coupled receptors (GPCRs) has suggested that internalization facilitates certain aspects of cellular signaling. Indeed, both epidermal growth factor- and β-adrenergic receptor-mediated activation of mitogen-activated protein kinase have been demonstrated to be dependent upon clathrin-mediated internalization [7,8]. However, Wnt signaling requires two kinds of receptors, the Frizzleds and the LRPs, and it would therefore be important to establish the cellular compartment where signaling takes place.

## Results

**Figure 1:**
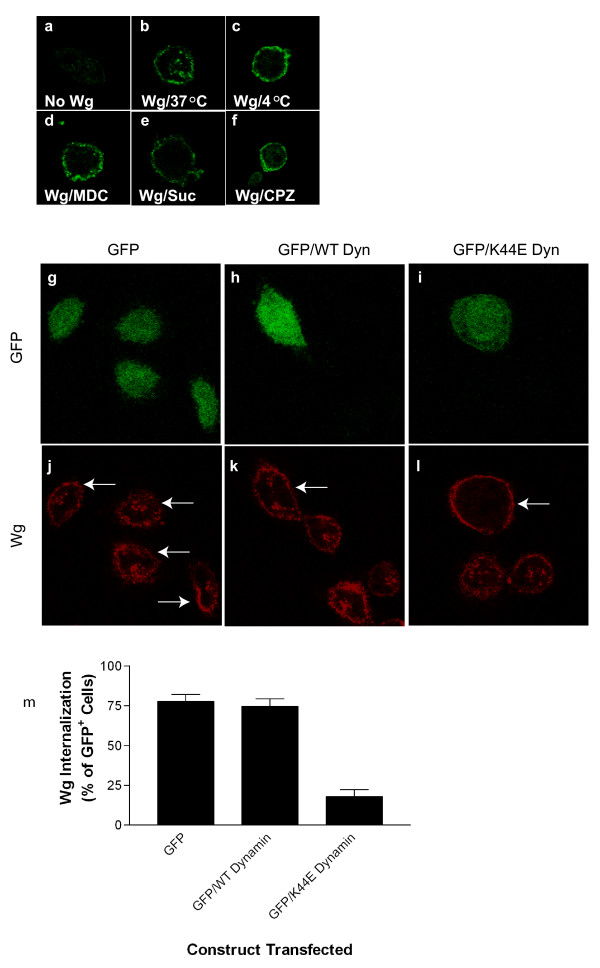
Wg internalizes by clathrin- and dynamin-mediated endocytosis, and localizes to transferrin recycling compartments. Immunostaining for Wg was performed in L cells incubated in the absence (**a**) or presence of Wg (**b-l, n-s**). Cells were incubated with Wg at 37°C (**b**) or 4°C (**c**), and in the presence of MDC (**d**), hypertonic sucrose (**e**), or CPZ (**f**). Internalized Wg was assessed by immunostaining. L cells transfected with GFP alone (**g **and **j **are identical fields; transfected cells indicated by arrows and non-transfected cells indicated by arrow-heads), or in combination with either wild-type dynamin (**h **and **k **are identical fields; transfected cells indicated by arrows and non-transfected cells indicated by arrow-heads) or K44E-mutated dynamin (**i **and **l **are identical fields; transfected cells indicated by arrows) were assessed for Wg endocytosis by immunostaining. (**m**) Quantitation of results in (**g-l**) where values represent means ± SEM from 3 independent determinations.

Here we have examined a potential role for endocytosis in Wnt signal transduction. Both Wnt-3A (a mammalian Wnt) and Wg (a *Drosophila *Wnt) stimulate high levels of β-catenin accumulation in L cells (Figure 3a), suggesting the utility of this cell line for dissecting the role of trafficking in the Wnt pathway. Incubation of L cells for 1 hour at 37°C with Wg-conditioned medium, but not control-conditioned medium (Figure 1a), leads to the appearance of a punctate immunostaining pattern as assessed by confocal microscopy (Figure 1b). Suggestive of internalization into vesicles, this punctate staining pattern is not observed when cells are incubated with Wg at 4°C (Figure 1c), a temperature known to inhibit vesicular trafficking pathways. We next sought to determine whether the internalization of Wnt is mediated by clathrin-coated vesicle formation, by far the most well-characterized mechanism for regulated endocytosis [9]. There are a variety of ways to perturb clathrin-mediated internalization, including the use of small molecules (e.g. monodansylcadaverine (MDC) and chlorpromazine (CPZ)), osmotics (e.g. hypertonic sucrose), dominant-interfering mutant proteins (e.g. dynamin), and loss of function reagents (e.g. siRNAs). MDC is believed to inhibit clathrin-mediated endocytosis by stabilizing nascent clathrin-coated vesicles and preventing uncoating [10], presumably reducing the availability of free clathrin to assemble at the plasma membrane. Hypertonic sucrose has been shown to prevent the proper assembly of clathrin lattices at the plasma membrane [11]. Moreover, CPZ blocks clathrin-mediated endocytosis through a mechanism whereby adaptor complex 2 (AP2) and clathrin are redistributed away from the plasma membrane, making clathrin unavailable for assembly at the cell surface [12]. Dynamin, a GTPase recruited to clathrin-coated pits, is an additional component of endocytosis which is thought to regulate the final step of vesicle biogenesis by catalyzing the scission of budding plasma membrane [9]. Mutation of lysine 44 (K44E) has been shown to decrease the affinity of dynamin for GTP, and expression of this mutant in cells dramatically inhibits endocytosis from the plasma membrane without affecting vesicular transport processes elsewhere in the cell [13]. Internalization of Wg is markedly reduced when cells are incubated in the presence of MDC (Figure 1d), hypertonic sucrose (Figure 1e), and CPZ (Figure 1f), with most of the staining in these cases being confined to the cell surface. Further, while L cells transiently transfected with either GFP alone or GFP in combination with wild-type dynamin internalize Wg into intracellular vesicles, cells expressing GFP in combination with the K44E mutant dynamin display predominantly cell surface staining (Figure 1g–l). By contrast, Wg internalizes quite robustly in adjacent non-transfected cells surrounding those harboring the K44E-mutated dynamin (Figure 1l). Whereas approximately 70–80% of cells transfected with GFP alone or in combination with the wild-type dynamin internalize Wg, only 20–25% of those expressing the K44E mutant protein exhibit comparable levels of endocytosis (Figure 1m). Consequently, the internalization of Wg into L cells occurs on a time-frame consistent with regulated endocytosis (minutes to an hour), and is sensitive to various manipulations which inhibit clathrin assembly and dynamin-mediated scission of budding plasma membrane.

**Figure 2:**
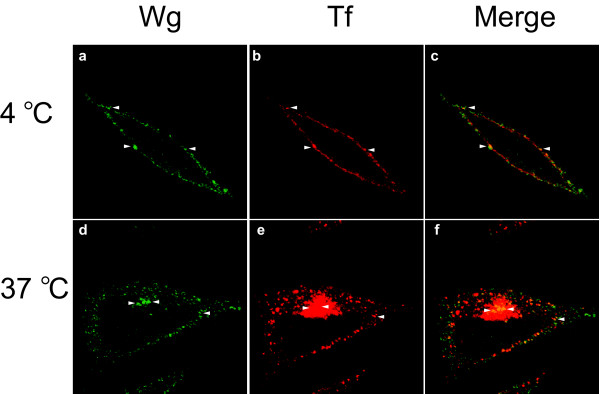
L cells were incubated simultaneously with Wg (green) and Cy3-conjugated transferrin (red) for 1 hour at 4°C (**a-c**) or 37°C (**d-f**). Arrow-heads indicate positions of Wg and transferrin co-localization.

**Figure 3:**
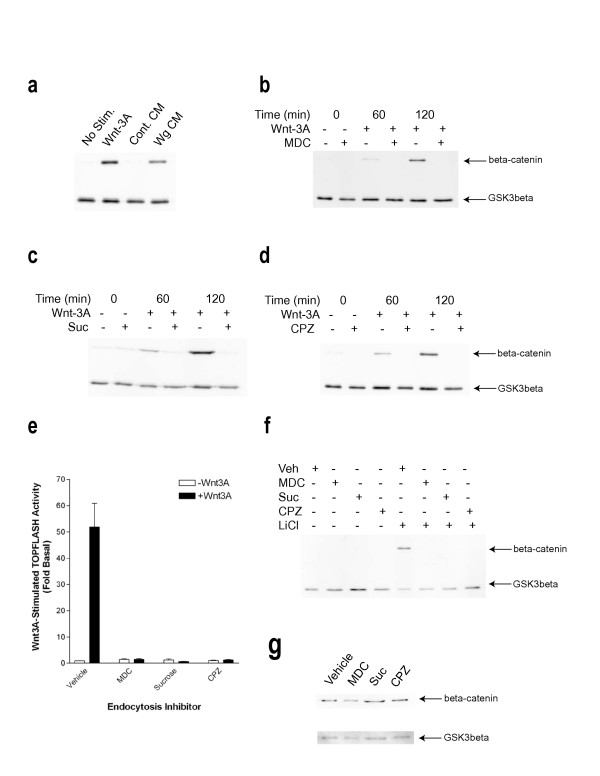
Wnt signaling is dependent upon clathrin-mediated endocytosis. (a) L cells were stimulated for 3 hours in the presence or absence of Wnt-3A (~100 ng/mL), control conditioned medium (Cont. CM), or Wg conditioned medium (Wg CM) and subsequently harvested and assayed for β-catenin and GSK3β levels by immunoblotting. β-catenin and GSK3β levels were similarly assessed in L cells stimulated with Wnt-3A over a 2-hour time-course in the presence or absence of MDC (300 μM) (b), hypertonic sucrose (0.45 M) (c), or CPZ (30 μM) (d). (e) LSL cells were stimulated for 5 hours in the presence or absence of Wnt3A with either DMSO (Vehicle), MDC, hypertonic sucrose, or CPZ and assayed for TOPFLASH reporter activity. Shown is a representative experiment from 3 independent determinations each performed in triplicate, in which values represent means ± SEM. (f) L cells were pre-incubated with or without DMSO Vehicle (Veh), MDC, hypertonic sucrose, or CPZ for 1 hour, followed by stimulation in the presence or absence of LiCl (50 mM) for 3 hours. Cells were subsequently harvested and assayed for β-catenin and GSK3β levels by immunoblotting. (g) SW480 cells were treated for 1 hour with Vehicle, MDC, hypertonic sucrose, or CPZ and were subsequently lysed under hypotonic conditions to enable measurement of cytosolic β-catenin and GSK3β levels.

We next sought to characterize the subcellular localization of internalized Wg. To identify potential intracellular compartments containing the trafficked Wnt, we performed Wg internalization assays in the presence of Cy3-conjugated transferrin. Receptors for transferrin constitutively cycle among the plasma membrane, early sorting endosomes, and perinuclear recycling endosomes, and have served as a classic system for the study of clathrin-mediated endocytosis [9,14]. Incubation of L cells with transferrin for 1 hour at 37°C leads to a steady-state distribution which is both punctate and perinuclear (Figure 2e and 2f), suggestive of the constitutive cycling of the protein from the plasma membrane via vesicles to perinuclear recycling endosomes. Internalized Wg exhibits partial overlap with transferrin, particularly in the perinuclear region of the cell (Figure 2d and 2f). By contrast, co-incubation of cells with Wg and transferrin at 4°C leads to an overlapping accumulation of the two proteins at the cell surface (Figure 2a and 2c). Hence, both Wg and transferrin not only internalize through similar endocytic pathways, but also have a similar steady-state localization in a perinuclear recycling endosome. The lack of complete co-localization of Wg and transferrin suggest that while the two proteins enter the cell through similar compartments, they ultimately have somewhat distinct fates. Indeed, it has previously been demonstrated that Wg transits through multivesicular bodies to the lysosomes for degradation following signal transduction [5].

Given that Wg is internalized within a time-frame relevant to Wnt signaling in L cells, we sought to determine whether the two processes are functionally coupled. Wnt signaling is typically measured through Wnt-stimulated accumulation of cytoplasmic β-catenin, as well as downstream target gene expression. L cells express very low basal levels of β-catenin and are quite responsive to exogenous mammalian Wnt-3A and *Drosophila *Wg (Figure 3a), making them a powerful system for measuring Wnt-induced increases in β-catenin. We assayed Wnt-stimulated accumulation of β-catenin in the presence or absence of the same mechanistically-distinct inhibitors which abolish Wg endocytosis. As demonstrated in Figure 3a, both Wnt-3A and Wg induce substantial stabilization of β-catenin over a 3-hour time-course, without altering the levels of a control cytoplasmic protein (GSK3β). By contrast, incubation of cells with MDC (Figure 3b), hypertonic sucrose (Figure 3c), and CPZ (Figure 3d) abrogates the Wnt-3A-stimulated increase in β-catenin. However, none of these inhibitors alter the levels of the control protein GSK3β. To assess the potential requirement for endocytic trafficking of the more downstream process of Wnt target gene expression, we measured Wnt-3A-stimulated expression of a luciferase reporter fused to a promoter containing TCF/LEF binding sites ("TOPFLASH"). As depicted in Figure 3e, vehicle-treated L cells stably expressing reporter constructs yield an approximately 50-fold increase in TOPFLASH activity relative to co-expressed LacZ following a 5-hour stimulation with Wnt-3A. By contrast, pre-incubation of these cells with MDC, hypertonic sucrose, or CPZ completely abolishes Wnt-induced reporter gene activity (Figure 3e). To order the endocytic trafficking step in the pathway, we assessed the sensitivity of various downstream pathway activators to these endocytosis inhibitors. Activation of the pathway through lithium-mediated inhibition of GSK3β is completely impaired when endocytosis is blocked (Figure 3f), a surprising observation which will be discussed later in this report. By contrast, the constitutive expression of cytoplasmic β-catenin in SW480 cells, which harbor an inactive version of APC, is resistant to perturbation of endocytosis (Figure 3g). Taken together, these results suggest that Wnt signaling is heavily dependent upon clathrin-mediated endocytosis, and that the explicit internalization event occurs between the steps of GSK3β inhibition and APC inactivation.

**Figure 4:**
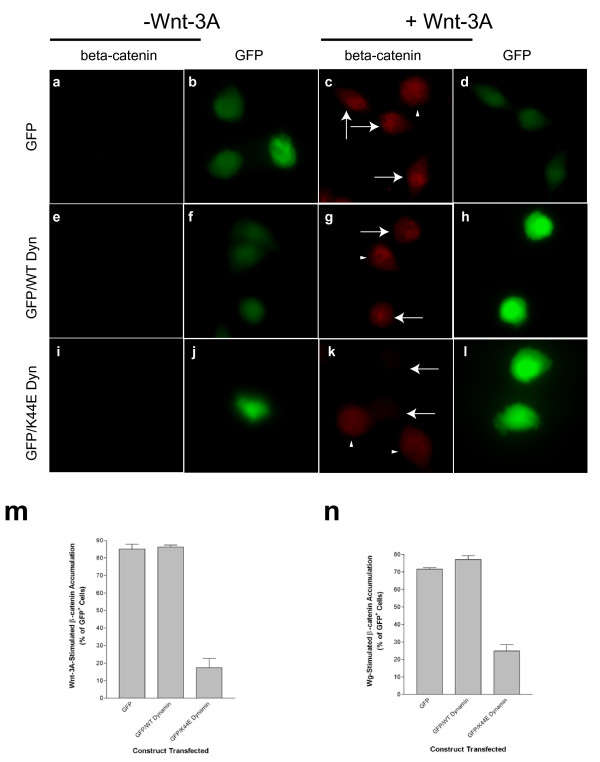
Wnt-stimulated accumulation of β-catenin is dependent upon dynamin activity. β-catenin levels were measured by immunostaining in L cells incubated in the presence (**c, d, g, h, k **and **l**) or absence (**a, b, e, f, i **and **j**) of Wnt-3A (~100 ng/mL) for 3 hours. L cells were transfected with either GFP alone (**a-d**) or GFP in combination with wild-type dynamin (**e-h**) or K44E-mutated dynamin (**i-l**). Arrows indicate transfected cells and arrow-heads indicate neighboring non-transfected cells. (**m**) Quantitation of immunofluorescence results from (**a-l**), expressed as the percentage of GFP-positive cells which are positive for β-catenin staining. (**n**) Quantitation of immunostaining experiments measuring Wg-stimulated (data not shown) accumulation of β-catenin in L cells transfected with the same constructs as in (**a-l**). In (**m**) and (**n**), values represent means ± SEM from 3 independent determinations in which approximately 30–70 cells were examined per condition.

In addition to perturbing clathrin assembly at the plasma membrane, we also examined the effect of the dynamin mutant on Wnt signaling. We measured both Wnt-3A- and Wg-stimulated accumulation of β-catenin on a cell-by-cell basis by immunofluorescence microscopy (Figure 4). Wnt-3A-stimulated β-catenin stabilization was measured in L cells transfected with GFP alone (Figure 4a–4d) or GFP in combination with either wild-type (Figure 4e–4h) or K44E-mutated dynamin (Figure 4i–4l). As observed in the western blot experiments (Figure 3), very little β-catenin is detected in the L cells in the absence of Wnt-3A (Figure 4a,4e,4i). Addition of Wnt-3A, by contrast, leads to prominent immunostaining of nuclear β-catenin in cells expressing GFP alone or in combination with wild-type dynamin (Figure 4c and 43 g where transfected cells are indicated by arrows and non-transfected cells by arrow-heads). However, as demonstrated in Figure 4k, Wnt-3A-stimulated accumulation of β-catenin is completely abolished in cells expressing the K44E-mutated dynamin (identified by GFP in Figure 4l and by arrows in Figure 4k), whereas neighboring non-transfected cells stabilize β-catenin comparably to control cells (Figure 4k where non-transfected cells are indicated by arrow-heads). As depicted in the quantitations (Figure 4m–4n), cells expressing GFP alone or in combination with wild-type dynamin demonstrate Wnt-dependent increases in β-catenin in approximately 70–90% of cells, with only approximately 20–30% of the K44E dynamin-expressing cells conferring a similar level of responsiveness. Taken together with the previous results using MDC, hypertonic sucrose, and CPZ, the ability of K44E-mutated dynamin to block Wnt signaling suggests that endocytosis is a key mechanistic step in the pathway.

**Figure 5:**
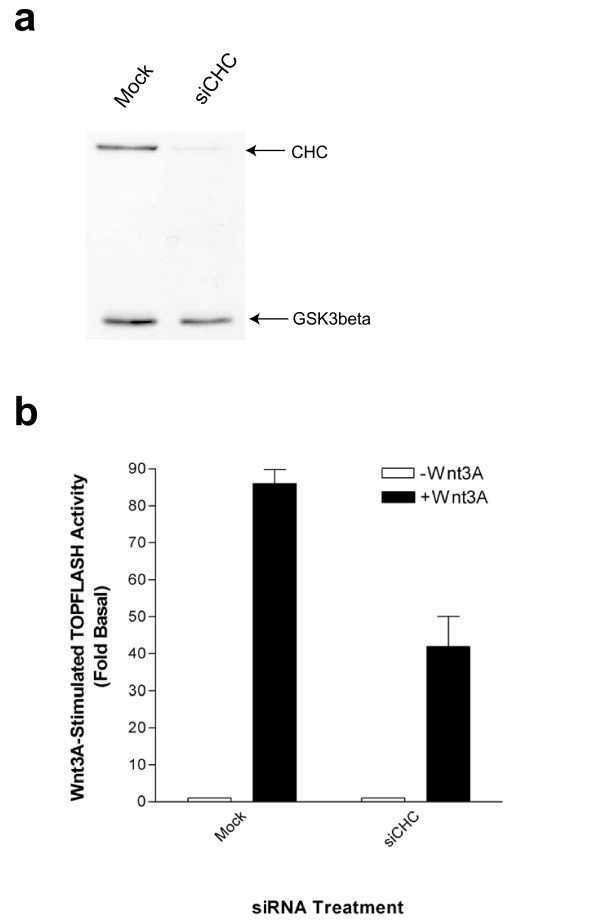
SiRNA-mediated depletion of clathrin impairs Wnt3A-stimulated reporter gene expression. LSL cells were transfected in two rounds with either empty plasmid vector (Mock) or siRNA corresponding to clathrin heavy chain (siCHC). (**a**) Levels of clathrin heavy chain and GSK3β were assessed by immunoblotting. (**b**) Cells were stimulated with purified Wnt-3A (~100 ng/mL) for approximately 8 hours, followed by lysis of cells and measurement of TOPFLASH and LacZ activities. Values represent means ± SEM from a representative experiment from 8 independent determinations.

The various methods used to inhibit clathrin-mediated endocytosis, including small molecules, osmotics, and dominant interfering mutants, all potently abolish Wnt internalization and signal transduction. In order to further examine the requirement of internalization in Wnt signaling, we have performed loss of function experiments using siRNAs directed against clathrin heavy chain. Although clathrin is composed of both light and heavy chains, it is the latter which is truly critical for establishing the lattice coat necessary for membrane budding, as siRNA-mediated silencing of this subunit has previously been shown to greatly attenuate transferrin endocytosis [15]. Consequently, we generated siRNA oligonucleotides corresponding to the region of clathrin heavy chain which has previously been shown to silence the protein [15]. As illustrated in Figure 5a, siRNAs against clathrin heavy chain selectively ablate this protein without affecting levels of the control protein GSK3β. Moreover, siRNA-mediated silencing of clathrin heavy chain substantially attenuates Wnt-3A-stimulated reporter activity (Figure 5b).

## Discussion

We demonstrate an essential role for endocytic trafficking in Wnt signaling. Through the use of various small molecule, dominant-interfering, and loss of function inhibitors of distinct stages of endocytosis, we define an explicit requirement for internalization in Wnt signaling. Our observation that β-catenin stabilization by both mammalian (Wnt-3A) and *Drosophila *(Wg) Wnt proteins is sensitive to blockade of endocytosis suggests that this requirement may be universal for the Wnt pathway, and not merely limited to one particular Wnt. After submission of this manuscript, Seto and Bellen [16] showed that Wg signaling in vivo is also dependent on internalization. The differential sensitivity of β-catenin stabilization to blockade of endocytosis in L cells treated with lithium and the APC mutant SW480 cells indicates that the key endocytic step may reside between GSK3β and APC in the pathway. Indeed, it was quite surprising that the stabilization of β-catenin effected by lithium stimulation (presumably through inhibition of GSK3β) required an intact endocytic pathway, given traditional models of the Wnt pathway scheme. However, recent studies have implicated GSK3β in the regulation of endocytic trafficking, perhaps independent of its role in the Wnt pathway [17,18]. Doronin et al. [17] demonstrates that stimulation of cells with lithium causes rapid endocytosis of the β_2_-adrenergic receptor, and Pelkmans et al. [18] report that siRNA-mediated silencing of GSK3β causes internalized transferrin to accumulate in early endosomes. Incorporating these recent findings [17,18] with our epistasis data could suggest the following scenario: inhibition of GSK3β in response to a Wnt signal (or directly via lithium) facilitates rapid internalization of the signaling machinery in a manner which sequesters it from the APC protein, thereby leading to the stabilization and nuclear translocation of β-catenin.

Over the past few years, a number of studies have suggested that endocytosis may have a facilitating role in signal transduction. For example, activation of Erk1/2 directly through RTKs, or through certain GPCRs, has been shown to require endocytosis [19,20,8]. Indeed, activated RTKs and GPCRs have been found to be present on populations of clathrin-coated vesicles containing a number of intermediate proteins in the Erk1/2 pathway including Ras, Raf, MEK, and Shc [21,19]. Further, it was recently discovered that clathrin-mediated endocytosis and subsequent trafficking of TGFβ receptors to endosomes is required for productive signaling [22]. These studies have contributed to a "signaling endosome hypothesis," suggesting that this organelle may serve as a nexus for a number of signal transduction pathways. Indeed, partial co-localization of internalized Wnt with transferrin further implicates the clathrin-mediated pathway in this process and suggests that a perinuclear recycling endosome functions as a compartment for Wnt signal transduction.

In contrast to the RTK, GPCR, and TGFβ pathways, very little is known about the subcellular localization of Wnt signaling. The few studies examining the potential endocytosis and trafficking of Wnt proteins have considered this as a mechanism for the formation of the Wnt morphogen gradient in the embryo or the termination of the pathway [5,6]. To date, no previous studies have suggested that internalization is explicitly required for propagation of Wnt signaling cascades. In an elegant study in the *Drosophila *embryo, a horseradish peroxidase-conjugated Wg was shown to be degraded by receptive cells in a manner sensitive to chloroquine and dependent upon *deep orange *, suggesting that the ligand is trafficked to the lysosomes [5].

## Conclusion

Taken together with our results, these data indicate that clathrin-mediated endocytosis may serve two purposes: promotion of Wnt signaling in an endosome, followed by ligand degradation and receptor down-regulation in the lysosomes. The critical balance between an endosomal localization conducive for productive Wnt signaling and lysosomal trafficking for attenuation of the signal may involve an explicit intracellular sorting step. Nevertheless, in the context of previous work on the internalization of Wg [5] and Wnt-5A [6], our study delineates a previously unappreciated role for endocytosis in Wnt signal transduction.

## Methods

### Materials

Monoclonal antibodies directed against β-catenin, GSK3β, and clathrin heavy chain were purchased from BD Pharmingen. The monoclonal anti-Wg (4D4) antibody was from the Developmental Studies Hybridoma Bank. Horseradish peroxidase (HRP)-conjugated secondary anti-mouse antibodies were obtained from Santa Cruz Biotechnology. MDC, sucrose, CPZ, and human holotransferrin were obtained from Sigma. Cy3 labeling reagent was obtained from Amersham Biosciences. Phosphate-buffered saline (PBS) was obtained from Invitrogen. Complete Protease Inhibitor cocktail tablets were obtained from Roche. Enhanced chemiluminescence (ECL) reagents were obtained from Perkin-Elmer. Polyfect was from Qiagen and Lipofectamine 2000 from Invitrogen.

### Cell lines and transfection

Murine L cells were obtained from American Type Cell Culture and cultured in Dulbecco's modified Eagle's Medium supplemented with 10% fetal bovine serum, penicillin/streptomycin, and glutamine. Cells were cultured in a humidified incubator at 37°C with 7% CO_2_. Cell lines were transfected with the pEGFP vector (Clontech) alone or were co-transfected with either wild-type or K44E-mutated dynamin I. Transfections were performed in 6-well dishes with a total of 1.5 μg of DNA using Polyfect reagent according to manufacturer's instructions, using a 10-fold excess of dynamin DNA relative to pEGFP DNA.

### Endocytosis assay

Internalization of Wg into murine L cells was measured by confocal microscopy. L cells were plated on round coverslips and were pre-incubated with either vehicle or inhibitors at 24-hours post-plating. In cases where dynamin constructs were used, GFP was co-transfected in order to identify the transfected cell population. Cells were incubated with either control conditioned medium or Wg conditioned medium for 1 hour at 4°C, followed by transfer of cells to 37°C for an additional 1-hour incubation. In Figure 1, 4°C conditions represent cells maintained at this temperature for the second hour of the experiment. In cases where chemical inhibitors of endocytosis were used, cells were first pre-incubated with inhibitors at 37°C for 1 hour, followed by incubation with Wg at 4°C for 1 hour, followed by incubation at 37°C for an additional 1 hour. Cells were washed 2X with ice-cold PBS supplemented with CaCl_2 _and MgCl_2 _(1 mM each) ("PBS++"), followed by fixation at room temperature with 4% paraformaldehyde in PBS. Cells were washed 3X for 5 minutes with PBS++, followed by permeabilization in PBS++ supplemented with 0.1% Triton X-100 ("Washing/Permeabilization Buffer") for 15 minutes at room temperature. Subsequently, cells were blocked for 1 hour at room temperature with PBS++ supplemented with 0.1% Triton X-100 and 5% normal donkey serum ("Blocking Buffer"). Cells were incubated with anti-Wg (4D4) antibody (diluted 1:50 in Blocking Buffer) overnight at 4°C, followed by three 15-minute room-temperature washes with Washing/Permeabilization Buffer. Wg immunostaining was detected using either a Cy3- or Alexa Fluor 488-conjugated anti-mouse secondary antibodies in blocking buffer at room temperature for 1 hour, followed by three 15 minute room-temperature washes with Washing/Permeabilization Buffer. Coverslips were mounted using Fluoromount G and imaged on a confocal Zeiss Laser Scanning Microscope 5 using the 63× objective with oil immersion. In cases where endocytosis with transferrin was examined, holotransferrin was labeled with Cy3 according to manufacturer's instructions, followed by separation from unincorporated dye using Sephadex G-50 gel-filtration chromatography. The Cy3-transferrin was incubated with cells at ~10 μg/mL in the presence or absence of Wg.

### β-catenin stabilization

Wnt-3A was purified from media conditioned from Wnt-3A-expressing L cells as previously described [23]. β-catenin accumulation in L cells was measured both by immunoblot and immunocytochemistry. Briefly, murine L cells in 6-well tissue culture plates were pre-incubated with either Vehicle (DMSO at 0.33%), MDC (300 μM), hypertonic sucrose (0.45 M), or CPZ (30 μM) at 37°C for 1 hour, followed by stimulation with purified Wnt-3A (~100 ng/mL) for 2–3 hours at 37°C in the continued presence of vehicle or inhibitors. Following Wnt stimulation, the medium was aspirated and the cells were washed with ice-cold PBS. The cells were subsequently scraped into 10 mM Tris-HCl/100 mM NaCl/1% Triton X-100/1X protease inhibitor cocktail/pH 7.4 and solubilized by rotation at 4°C for 20 minutes. Lysates were clarified by refrigerated micro-centrifugation at 20000 × *g *for 20 minutes. In contrast to L cells, SW480 cells were swelled on ice in 10 mM Tris-HCl/0.2 mM MgCl_2_/0.25 M sucrose/1 mM EDTA/pH 7.4, followed by lysis by 20 strokes in a tight-fitting Dounce homogenizer. Lysates were first centrifuged at 1000 × *g *for 10 minutes at 4°C to pellet nuclei, debris, and unlysed cells. Supernatants from this spin were further clarified by refrigerated micro-centrifugation at 20000 × *g *for 1 hr. Supernatant from the micro-centrifugations was subsequently added to SDS-PAGE sample buffer and electrophoresed on a 10% gel. Transfer to nitrocellulose was performed at 12 V (constant current) for 40 minutes. Blots were blocked in PBS/0.1% Tween-20/5% non-fat dried milk ("Blotting Buffer") for 10 minutes, followed by incubation with a cocktail of primary anti-β-catenin and anti-GSK3β antibodies in Blotting Buffer overnight at 4°C with agitation. Blots were washed extensively with Blotting Buffer, followed by incubation with HRP-conjugated anti-mouse secondary antibodies in Blotting Buffer for 1 hour at room temperature. Following extensive washing in Blotting Buffer and PBS, immunoreactivity was assessed by ECL.

For measurement of β-catenin by immunofluorescence, L cells were plated on round coverslips in 12-well dishes and allowed to adhere overnight. 24-hours post-plating, L cells were pre-incubated and stimulated in the same manner as previously described for the immunoblot analysis. In cases where the dynamin constructs were used, cells were co-transfected with GFP to mark the transfected cell population. Wnt stimulations were performed 48 hours post-transfection. Following stimulation, the medium was aspirated and cells were gently rinsed twice with PBS++. Cells were subsequently fixed in 4% PFA in PBS for 15 minutes at room temperature, followed by three 5-minute washes in PBS++. Cells were permeabilized in Washing/Permeabilization Buffer for 15 minutes at room temperature. Permeabilization of the nuclear envelope was accomplished by a 10-second incubation with room-temperature methanol, followed by three 5-minute washes in PBS++. Cells were blocked for 1 hour at room temperature with Blocking Buffer. Incubation of primary anti-β-catenin antibody (diluted 1:100 in Blocking Buffer) was overnight at 4°C. Primary antibody was aspirated and cells were given three 15-minute washes with Washing/Permeabilization Buffer, followed by incubation with Cy3-conjugated secondary anti-mouse antibody in Blocking Buffer for 1 hour at room temperature. Secondary antibody was aspirated and cells were given three 15-minute washes with Washing/Permeabilization Buffer before mounting the coverslips in Fluoromount G. Immunostaining experiments were observed with a Zeiss Axioplan 2 microscope using the 40× objective.

### Measurement of TCF/LEF activity

L cells stably harboring the TOPFLASH and LacZ constructs ("LSL cells") were plated in 96-well microtiter dishes to approximately 80–90% confluency. 24 hours post-plating, LSL cells were pre-incubated for 1 hour in the presence of either Vehicle (DMSO at 0.33%), MDC (300 μM), sucrose (0.45 M), or CPZ (30 μM). Subsequently, cells were stimulated with purified Wnt-3A (~100 ng/mL) for 5 hours at 37°C in the continued presence of vehicle or inhibitors. Expression of luciferase was measured in a Lumat LB 9507 luminometer (Berthold) using the TROPIX kit according to manufacturer's instructions. All assays were performed in triplicate, and the relative luciferase units were normalized to LacZ readings.

### siRNA targeting of endocytic proteins

siRNAs directed against clathrin heavy chain were synthesized in the Stanford Protein and Nucleic Acid Facility. Sequences for clathrin heavy chain (sense: 5'-GCAAUGAGCUGUUUGAAGATT-3'; anti-sense: 5'UCUUCAAACAGCUCAUUGCTT-3') which were identical in human and murine isoforms of the proteins were selected from siRNAs described previously.(Huang et al., 2004) Using Lipofectamine 2000, siRNAs (~2 μg) were transfected along with empty pcDNA3 or pEGFP vector (2 μg) into LSL cells in 6-well plates, followed by a second round of transfections 24 hours later. Approximately 12 hours after the second transfection, the LSL cells were split into fresh media in preparation for reporter assays (either 24 well or 96 well plates) and harvesting for immunoblots. In reporter experiments, cells were incubated with purified Wnt-3A (~100 ng/mL) for approximately 8 hours, followed by measurement of TOPFLASH and LacZ activities.

## Authors' contributions

JB conceived of and executed the experiments in this study. RN provided advice with design of experiments and interpretation of results, as well as funding for this work.
